# Acute Toxicity of Cu-MOF Nanoparticles (nanoHKUST-1) towards Embryos and Adult Zebrafish

**DOI:** 10.3390/ijms22115568

**Published:** 2021-05-25

**Authors:** Natalia Abramenko, Gregory Deyko, Evgeny Abkhalimov, Vera Isaeva, Lyubov Pelgunova, Eugeny Krysanov, Leonid Kustov

**Affiliations:** 1 N.D. Zelinsky Institute of Organic Chemistry, RAS, Leninsky pr. 47, Moscow 119991, Russia; natalimsu@gmail.com (N.A.); gdeyko@gmail.com (G.D.); veraisaeva2019@mail.ru (V.I.); 2 A.N. Severtsov Institute of Problems of Ecology and Evolution, RAS, Leninsky Prospect, 33, Moscow 119071, Russia; lubo4ka007@bk.ru (L.P.); krysanov@sevin.ru (E.K.); 3 A.N. Frumkin Institute of Physical Chemistry and Electrochemistry, Russian Academy of Science, Leninsky Prospect, 31-4, Moscow 119071, Russia; abkhalimov@ipc.rssi.ru; 4 National Science and Technology University MISiS, Leninsky Prospekt 4, Moscow 119071, Russia; 5 Chemistry Department, Moscow State University, Leninskie Gory 1, bldg. 3, Moscow 119991, Russia

**Keywords:** HKUST-1, toxicology, zebrafish, uptake, MOF nanoparticles

## Abstract

Metal-organic frameworks (MOFs) demonstrate unique properties, which are prospective for drug delivery, catalysis, and gas separation, but their biomedical applications might be limited due to their obscure interactions with the environment and humans. It is important to understand their toxic effect on nature before their wide practical application. In this study, HKUST-1 nanoparticles (Cu-nanoMOF, Cu_3_(btc)_2_, btc = benzene-1,3,5-tricarboxylate) were synthesized by the microwave (MW)-assisted ionothermal method and characterized by X-ray powder diffraction (XRD) and transmission electron microscopy (TEM) techniques. The embryotoxicity and acute toxicity of HKUST-1 towards embryos and adult zebrafish were investigated. To gain a better understanding of the effects of Cu-MOF particles towards *Danio rerio* (*D. rerio)* embryos were exposed to HKUST-1 nanoparticles (NPs) and Cu^2+^ ions (CuSO_4_). Cu^2+^ ions showed a higher toxic effect towards fish compared with Cu-MOF NPs for *D. rerio*. Both forms of fish were sensitive to the presence of HKUST-1 NPs. Estimated LC_50_ values were 2.132 mg/L and 1.500 mg/L for zebrafish embryos and adults, respectively. During 96 h of exposure, the release of copper ions in a stock solution and accumulation of copper after 96 h were measured in the internal organs of adult fishes. Uptake examination of the major internal organs did not show any concentration dependency. An increase in the number of copper ions in the test medium was found on the first day of exposure. Toxicity was largely restricted to copper release from HKUST-1 nanomaterials structure into solution.

## 1. Introduction

Metal-organic frameworks (MOFs) are a new class of hybrid porous materials composed of metal ions and organic linkers connected via coordination bonds. Due to their unique structure, record-high surface area, and extremely high porosity, MOFs have a great perspective in various applications. MOF materials are widely studied for gas storage, separation, agriculture, geology, sensing applications, biomedicine, and catalysis [1,2,3]. Moreover, MOFs are considered promising materials for biomedical applications including developing more effective therapies [1,4,5,6,7,8,9,10,11]. The ability of MOF to accommodate multiple therapeutic agents, potential antimicrobial features of some MOFs [11,12,13] in combination with unique properties of NPs [14] make nanosized MOF particles better materials for drug delivery and integration in biomedicine [7].

Moreover, it was shown that MOF particles can be a good candidate for bactericidal application alone and as a part of composite materials resistant to bacterium strains [15,16]. A simultaneous effect of MOF-5 and silver NPs was observed for photocatalytic disinfection activity on *Escherichia Coli* bacteria-infected water [17]. The high durability and stability of MOF to the agglomeration and oxidation, and the release of ions from the MOF surface made MOF particles a promising candidate to act as a reservoir of metal ions with antibacterial properties [18]. Gradual ions’ release provides a sustained and prolonged antibacterial effect [19].

Information on the toxicity and biodegradation of MOF materials is mainly represented by in vitro tests on cell cultures. A zinc-containing nanoscale MOF has shown particle-induced cellular effects towards neural cells and demonstrated a time- and concentration-dependent toxicity on rat pheochromocytoma cells [20]. Preliminary toxicity assessment showed that the presence of metal atoms in MOFs network could influence the homeostasis of the exposed live system both extracellularly and intracellularly [21,22]. According to the research by Tamames-Tabar et al., the cytotoxicity of MOFs NPs depends on the nature of metal ions in their structure, as well as on the tested cell line [12]. It was supposed that the hydrophobicity of MOFs might play a critical role in their interactions with biological systems, due to a tendency to degradation and metal release. The same trend was observed in studies of the correlation between the MOF hydrophobicity and their toxicity towards human lung epithelial cells. It was shown that the hydrophobic ZIF-8 particles were more toxic than the hydrophilic MIL-160 particles [23] (IC_50_ values were 61 µg/mL and 433 µg/mL, respectively). At the same time, some of the observed MOF particles demonstrate moderate or high toxicity to a live system.

The toxicity of sixteen uncoated nano-sized MOFs was evaluated towards HepG2 and MCF7 cells and for some of them towards *D. rerio* [24]. Depending on the MOF’s structure and the stability in tested media, nanoscale MOFs demonstrate high, moderate, or no toxicity. According to their cytotoxic effect, nano-MOF particles were ranged from nontoxic or barely toxic (UiO-66, UiO-67, Co-MOF-74, Mg-MOF-74), moderately cytotoxic (ZIF-7, MIL-100, MIL-101), and highly toxic (ZIF-8, HKUST-1). The observed cytotoxic effect was mainly in good agreement with in vivo toxicity towards fishes [25]. In particular, ZIF-8 with the particle size 80 ± 15 nm and HKUST-1 with the size of 75 ± 28 nm were highly cytotoxic and show a rapid toxic effect on zebra fish embryos. The authors suggested that the toxic effect can be explained by the degradation of MOFs and releasing the metal ions from the structure. Similar results were found for ZIF-8 NPs (d = 103.95 nm) on zebrafish adults [25].

Low toxicity of Fe-MOF in vitro was confirmed by in vitro study with rats [26,27]. Taking into consideration the observed low cytotoxicity of porous MIL-100(Fe) particles towards different cell lines and its good stability, authors consider this Fe-MOF as a potential candidate as a drug carrier [13,24]. In agreement with the low in vitro toxicity, Fe-MOF NPs demonstrate slight in vivo toxicity to rats with rapid sequestration by the liver and spleen after intravenous administration [26]. All studied Fe-nanoMOFs were degraded and directly eliminated in urine or feces without metabolization and sights of substantial toxicity. Cu-MOF particles, on the opposite, demonstrate a highly toxic effect on cells [28] and animals [24]. On the contrary, the low stability of Cu-MOF particles in cell cultures and easy ions release were revealed in various research [24,28].

Thus, without a proper investigation of MOFs’ safety, the increasing potential uses of MOFs in biomedical applications may increase health risks to the patient. There is uncertainty related to the MOF’s hazard effect to live systems; a few data about their recycling and degradation are published [24,29]. Therefore, for the further and intensive application of MOF materials, it is critical to understand their toxic effect on nature and humans. There are some challenges and hindrances associated with the applications of MOFs: choice of an appropriate solvent, additives, and non-toxic organic linkers for sustainable production of eco-friendly MOFs. The toxic properties of MOFs should be clarified before further expansion of their practical applications.

Therefore, the purpose of this study was to assess the impact of Cu-containing MOF NPs on freshwater fishes, their uptake, and their effect on embryos. Herein, we evaluate the fate of HKUST-1 NPs Cu_3_(btc)_2_, btc = benzene−1,3,5-tricarboxylate) with the size of ~20–30 nm in a test medium, their accumulation in internal organs, and toxicity of Cu-MOF NPs (HKUST-1) towards zebrafish embryos and adult animals for supporting risk assessment of nanoMOF materials. The study compares the sensitivities of different forms of zebrafish species to Cu-MOF NPs and copper ions.

## 2. Results and Discussion

### 2.1. Characterization of the HKUST-1 Nanomaterials

**Scheme 1:**
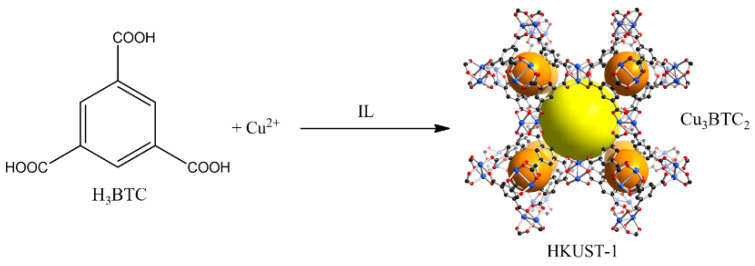
Synthesis of HKUST-1 (IL–1-ethyl-3-methylimidazolium bis(trifluoromethylsulfonyl)imide).

The HKUST-1 nanomaterial was synthesized by the MW-assisted technique at the atmospheric pressure according to Scheme 1 (see Section 3 for details).

**Figure 1:**
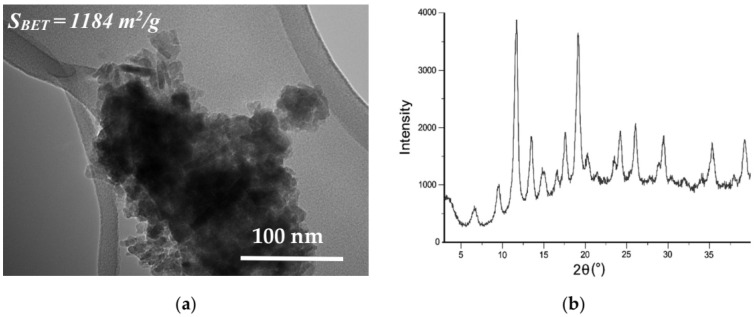
TEM micrograph (**a**) and PXRD pattern (**b**) of the HKUST-1 NPs synthesized under ionothermal conditions in the MW fields.

The nanoparticle size and structural characteristics for the prepared nanoHKUST-1 powder were estimated from TEM measurements and powder X-ray diffraction data (Figure 1).

The obtained HKUST-1 nanomaterial obtained in MW fields was composed of very small nanocrystals with a distorted prismatic shape with a size of ~20–30 nm integrated into the larger associates around 50–100 nm in size. The powder diffraction pattern of the HKUST-1 sample was typical for the cubic unit cell with *a* = 26.292 Å in the space group Fm-3m. Broadening of the reflexes in the PXRD pattern confirmed the small size of HKUST-1 nanoparticles. The specific surface area (BET) measured for the HKUST-1 nanomaterial is 1184 m^2^/g [30].

The estimated content of Cu in the obtained sample was 35.01 ± 0.29% by mass, this value was used for acute toxicity tests with zebrafish adults and embryos.

### 2.2. Toxicity Tests

**Figure 2:**
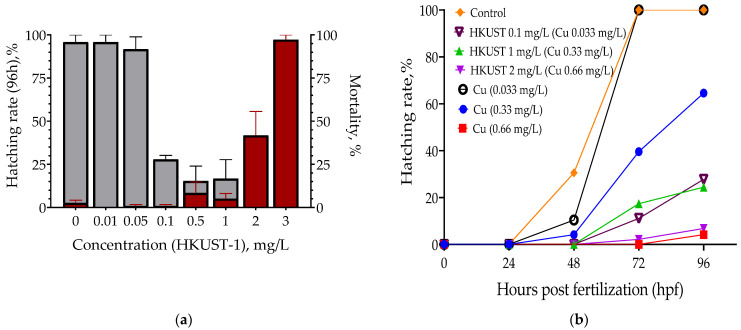
Mortality of *D. rerio* embryos (**a**) and hatching rate (**a**,**b**) for HKUST-1 (Cu-MOF) NPs and Cu^2+^ ions 0–96 h exposure.

Several series of experiments were performed to evaluate the working concentration range for the tested substance with *D. rerio* embryos. We observed a 100% mortality in the HKUST-1 solution with a high concentration and no effect in solutions with a low concentration of HKUST-1 NPs (Figure 2). The lowest observed effect concentration (10% mortality, LOEC) and half-lethal concentration (50% mortality, LC_50_) values were calculated in acute toxicity bioassays. The same experiments were carried out with zebrafish embryos and copper ions at the same concentrations as concentrations of Cu in HKUST-1 NPs (calculated from the Cu content).

Exposure of embryos to the HKUST-1 suspensions and copper sulfate solutions induced a range of mortality from 1% to 100% at 96 h. Copper ions appeared more toxic than Cu–MOF NPs.

**Figure 3:**
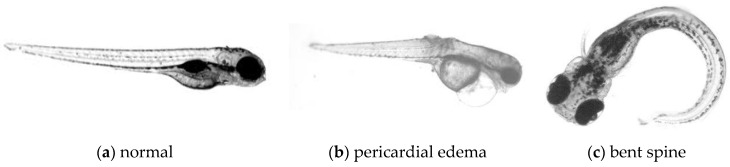
Representative images of zebrafish embryos (**a**) normal development; (**b**) pericardial edema and yolk-sac edema; (**c**) bent spine.

The hatching rates for the control group embryos were higher in comparison with the groups treated with Cu ions and HKUST-1 NPs. In the control groups of embryos, normal development was observed: all of the embryos hatched after 72 h. The hatching rate and malformation degree were in a clear concentration dependence from the exposure concentration. For all tested samples, there was a delay in the hatching rate for surviving embryos. Typical malformations included a shortened body length, bent spine, induced tail malformation, pericardial and yolk-sac edemas in zebrafish embryos (Figure 3).

Copper ions are considered the most toxic form of copper. It was shown that copper from Cu-MOF NPs affects the hatching rate, survival, and embryogenesis. Copper might cause osmotic disturbances and inactivate chorionase that finally affects the embryo’s activity and the muscular movements necessary to break the eggshell. It has also been associated with a typical malformation in hatching embryos such as low pigmentation of the body, spinal cord deformation, jaw underdevelopment, and decreased length.

Previously reported toxicity of nanoHKUST-1 (size: 75 ± 28 nm) for similar exposure conditions and species [24] is in good agreement with our results found for *D. rerio* embryos. Our results showed that the HKUST-1 suspension and Cu^2+^ ions were both toxic to *D. rerio* embryos.

We observed a more toxic effect of copper ions (sulfate) than the Cu-nanoMOF sample in the case of the mortality value. The inverse relationship was found in the case of the hatching rate. Copper can influence chorionase, cause osmotic disturbances, and strongly affects the hatching process. The delay of development was found for survived animals for both tested samples. The HKUST-1 samples demonstrated a more sufficient toxic effect for the hatching rate. This difference can be explained by the more complicated composition of the mixture and presence of the organic linker in the solution; as well as by the cumulative effect of the mixture on embryo development.

**Figure 4:**
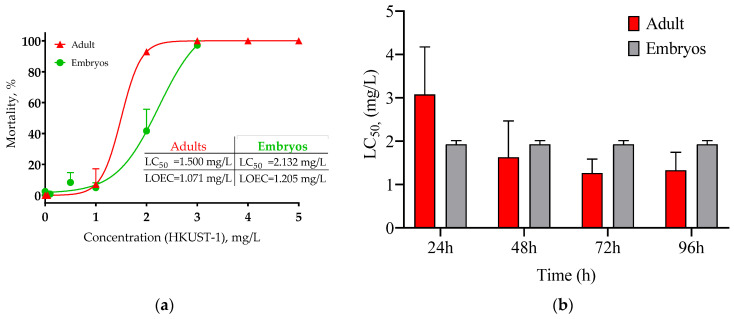
Mortality (**a**) and LC_50_ data (**b**) of HKUST-1 (Cu-MOF) particles in embryos and adult zebrafish over 96 h exposure.

Further research was focused on toxicity assay with zebrafish adult animals. In experiments with adult animals, the toxicity was explored in a wider concentration region in comparison to embryos to estimate the LC_50_ values (Figure 4).

Our results showed that the HKUST-1 suspension was toxic for both *D. rerio* adults and embryos starting with 24 h of exposure. The toxicity of the HKUST-1 suspension increased sharply after 24 h and change daily for adult zebrafish until 72 h. After 72 h, there were no significant changes in the LC_50_ values for adult animals (Figure 3b); the main mortality for embryos was at 24 h and did not change until the end of the experiment.

As the toxicity of Cu ions is well investigated, we used literature data for zebrafish adults. Additionally, adult fishes demonstrate a higher sensitivity in the case of copper ions. Compared with the Cu nanoMOF suspension, CuSO_4_ had higher toxicity in all fish forms with 96 h LC_50_ of 0.396 and 0.05–0.10 mg/L for zebrafish embryos and adults, respectively. The estimated 96 h LC_50_ values of nanoHKUST-1 and Cu^2+^ in zebrafish embryos were 0.746 mg Cu/L (2.132 HKUST-1 mg/L) and 0.396 mg/L, respectively. Summary data for Cu ions and HKUST-1 NPs are presented in Table 1.

In agreement with previous studies [32,33], we found that adult zebrafishes were more sensitive to copper than embryos. It should be emphasized that the fish development stage can significantly affect the response to copper, in line with other similar studies [31,32,33].

Copper is a critical trace element necessary for the normal growth and metabolism of many living organisms. However, it can be toxic at high concentrations. Fishes are very sensitive to the Cu presence: they are up to 10 to 100 times more sensitive to the toxicity of copper than mammals [34].

It is commonly accepted that gills are the primary target of the toxic effect of copper on fishes. The high copper concentrations may cause critical histopathological changes, ion regulatory disturbances, and as a consequence, rapid mortality. The presence of copper can result in hypoxemia and cause histopathological changes such as cell swelling and thickening of lamellae, congestion, epithelial detachment, lamellar synechiae, and fusion of lamellae. However, mortality might result from ionoregulatory disturbances that lead to elevated blood pressure and cardiovascular collapse. Moreover, there is a suggestion that the chorion may protect the embryo by trapping metal ions and prevent their transport across the chorion into the perivitelline fluid [35].

Mortality was induced in zebrafish after 96 h with the LOEC of the HKUST-1 suspension of 1.071 and 1.205 mg/L for adults and embryos, respectively. The test with benzene-1,3,5-tricarboxylic acid was performed to estimate the sensitivity of zebrafish to the organic component of the MOF structure. No significant mortality was observed in the experiment with the organic linker to both fish forms, only the highest concentration was a bit toxic for *D. rerio* embryos (data are not shown).

**Figure 5:**
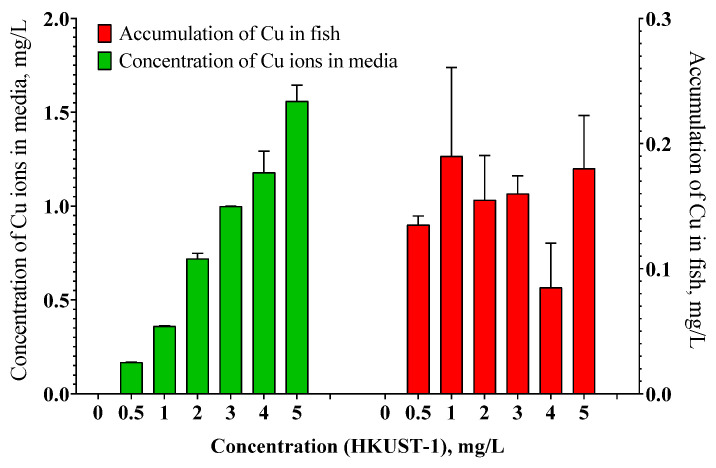
Accumulation of copper in internal organs of zebrafish adults (liver, spleen, gastrointestinal tract) after 24 h of exposure (red) and copper ions concentration in water at the end of the experiment (green). Results expressed as mean ± standard deviation of duplicate tests.

The toxic effect of Cu nano-MOF was evaluated by testing the Cu accumulation in main internal organs at the end of experiments. Accumulation of Cu was assessed in the gastrointestinal tract, liver, spleen as a combined sample. The cumulative samples were taken from all fish animals in the tested and control groups. The data are presented in Figure 4. The tissue sample (taken from fish, alive or not) was dissolved in nitric acid (1:1) in a MARS high-pressure microwave oven and analyzed. Moreover, exposure concentrations were measured during the experiment in the supernatant to estimate the possibility of Cu ions released from the MOF surface (Figure 5).

HKUST-1 NPs were found to demonstrate a tendency to dissolution in the testing water. On the first day of the experiment, practically all Cu ions from the HKUST-1 sample were dissolved in the zebrafish water medium. There was no significant accumulation of Cu in the internal organs of the treated fish animals, only at the highest concentration a slight increase in the concentration of copper was observed.

The amount of Cu in eggs water and tested media can suggest fast degradation of HKUST-1 with the release of ions in aquatic media. These findings suggest that the major feature of Cu-nanoMOF toxicity can be associated with the ion release from the HKUST-1 NPs.

## 3. Materials and Methods

Copper(II) nitrate trihydrate (98% purity), benzene-1,3,5-tricarboxylic acid (H_3_btc, 95% purity), 1-ethyl-3-methylimidazolium chloride (98% purity), lithium bis(trifluoromethylsulfonyl) imide (99% purity), and *N*,*N*′-dimethylformamide (DMF, 99.8% purity) were purchased from Acros (Thermo Fisher Scientific, Geel, Belgium, EU). *N*,*N*′-dimethylformamide (DMF) was distilled over CaH_2_ under reduced pressure.

Synthesis of ionic liquid 1-ethyl-3-methylimidazolium bis(trifluoromethylsulfonyl)imide (EMIM[Tf_2_N]) was carried out according to a modified literature procedure [36]. The solution of 1-ethyl-3-methylimidazolium chloride (14.2 g, 0.1 mol) in 25 mL of distilled water was mixed with a solution of lithium bis(trifluoromethylsulfonyl)imide (28.7 g, 0.1 mol) in 50 mL of distilled water and 100 mL of dichloromethane in a separation funnel. The organic layer was separated and then washed with small portions (5–10 mL) of distilled water until a negative reaction of the water layer with AgNO_3_. The obtained dichloromethane solution of ionic liquid was concentrated using a rotary evaporator and then dried in a vacuum (10^−2^ Torr) overnight at 80 °C.

### 3.1. Synthesis of Nano-MOF Particles

Nanosized HKUST-1 sample. Synthesis of the nanoHKUST-1 material was carried out by the MW-assisted ionothermal technique using EMIM[Tf_2_N] (1-ethyl-3-methylimidazolium bis(trifluoromethylsulfonyl)imide) as the only solvent. Then, 8.58 mmol of Cu(NO_3_)_2_ 3H_2_O and 4.76 mmol of H_3_btc was mixed with 10.36 g of EMIM[Tf_2_N], and the mixture was transferred into a glass ampoule and heated at atmospheric pressure in a chamber of an MW oven “Vigor” (200 W, 1.5 min, 125 °С). The blue crystalline product was centrifuged and rinsed with DMF (4 × 10 mL) and ethanol (2 × 10 mL). Then the nanoHKUST-1 material was dried under a vacuum (130 °C, 8 h, 10^−2^ Torr).

The nanoHKUST-1 suspension was freshly prepared by dispersing the HKUST-1 powder in standardized synthetic fresh water and sonicated for 5 min with a probe sonicator (Cole Parmer CPX 130 ultrasonic processor). The standardized synthetic freshwater consisted of 0.29 g/L NaCl, 0.013 g/L KCl, 0.0365 g/L CaCl_2_, 0.815 g/L MgCl_2_ × 6H_2_O; pH = 7.2–7.3.

### 3.2. Characterization

The synthesized HKUST-1 samples were characterized by powder X-ray diffraction and scanning electron microscopy (SEM).

X-ray powder diffraction data were collected in reflection mode using an EMPYREAN instrument (PANalytical, Malvern, United Kingdom) equipped with a linear X’celerator detector and non-monochromated Cu-Kα radiation (α = 1.5418 Å), measurement parameters: tube voltage/current 40 kV/35 mA, divergence slits of 1/16 and 1/8°, 2 θ range 5–50°, speed 0.1° min^−1^.

TЕM analysis. The microstructure and nanoparticle size of Cu-MOF were studied using a SU8000 field-emission scanning electron microscope (FE-SEM, Hitachi, Mannheim, Germany). Images were acquired in the second electron mode by an upper detector at an accelerating voltage of 10 kV. The target-oriented approach was utilized for the optimization of the analytical measurements [37].

Elemental analysis. Elemental qualitative and quantitative analysis of tissue samples was performed according to ISO/TS 18507:2015 guideline [38]. Elemental analysis was carried out by total external reflection (TXRF) X-ray method using an S2 PICOFOX spectrometer (Bruker, AXS, Germany). The sample was dissolved in nitric acid (1:1) in a MARS high-pressure microwave oven (CEM Corporation, USA). “Natural” spectra of the samples were taken to select the internal standard. Gallium (Ga) was selected as the internal standard for the series of samples. A standard Ga solution (Gallium nitrate solution GSO 7641–98, GDVI.410408.024.PS) was introduced into each sample at the rate of 1 mg/L of the initial liquid. The obtained mixture was mounted onto special quartz disks, dried at a temperature of 60 °C, the probe then was placed in an S2 PICOFOX instrument.

### 3.3. Ecotoxicity Testing

Experiments with zebrafish (*D. rerio, Hamilton 1822*) embryos were carried out according to the OECD guideline [39]. Fertilized zebrafish eggs were obtained from an AB wild-type zebrafish. Adult zebrafishes were maintained under standard conditions in aquaria with the volume 20 L at 26 °C in a 14 h light, 10 h dark cycle. The eggs were collected immediately after spawning; the quality was estimated under a stereomicroscope. The embryos were incubated in the embryo media: 0.29 g/L NaCl, 0.013 g/L KCl, 0.0365 g/L CaCl_2_, 0.815 g/L MgCl_2_ x 6H_2_O; pH = 7.2–7.3 in 24 well plates (Chimmed Group, Moscow, Russia). Embryos were kept in an incubator at T = 28 °C with photoperiod 14 h light/10 h dark. Throughout the experiment, the replacement of the medium was not performed.

The HKUST-1 suspension was freshly prepared by dispersing the HKUST-1 powder in standardized synthetic freshwater and sonicated for 5 min with a probe sonicator (Cole Parmer CPX 130 ultrasonic processor). The standardized synthetic freshwater consisted of 0.29 g/L NaCl, 0.013 g/L KCl, 0.0365 g/L CaCl_2_, 0.815 g/L MgCl_2_ × 6H_2_O; pH = 7.2–7.3.

The embryo development stages were examined using a stereomicroscope Carl Zeiss, Stemi 2000 C (Carl Zeiss Microscopy GmbH, Germany). Embryonic stages were matched according to Kimmel et al., 1995 [40]. An acute exposure regime of 96 h was used, from 0 h post-fertilization (hpf) to 96 hpf, including the major stages of embryogenesis. At the stage of a blastula, zebrafish embryos were transferred in 2 mL of freshly prepared solutions containing tested samples of HKUST-1 suspensions and a control solution, 1 embryo per well into 24-well plates. Embryos were tested daily using a Stereo Microscope Zeiss Stemi 2000. The number of dead embryos, embryos with developmental disorders, and the number of hatched larvae were counted. The stages of development of eggs were monitored under a binocular microscope Carl Zeiss, Stemi 2000 C (Carl Zeiss Microscopy GmbH, Germany). We checked such malformations of zebrafish embryos as pericardial edema, scoliosis, and a decrease in the body size. At the end of the experiment, the mortality index in the population, the percentage of morphological abnormalities in the zebrafish embryos, hatching rate, and morphological variation among survivors were determined. Malformations and other teratogenic effects were scored. The hatching success of zebrafish embryos was determined. The survival rate, the percentage of malformed embryos, and the rate of hatching were calculated at 96 hpf. During the experiment, the mortality of the embryos, the hatching rate, and the morphological variations among survivors was determined.

Five concentrations of nano-MOF suspensions in five replicates were used and compared with the toxicity of CuSO_4_ (copper ions). The concentration-response curve of copper sulfate was applied in the case of *D. rerio* embryos to assess to what degree the toxicity of the Cu-nanoMOF particles could be accounted for by the copper ions released from the MOF surface.

The exposure studies with adult fishes were carried out according to the OECD guidelines 236 [41]. Three-month aged zebrafishes (length: 26.1 ± 0.5 mm, weight: 0.16 ± 0.01 g) were used for the exposure. Seven fishes were randomly sited into the exposure in 1.5-L tanks. The system was aerated to maintain the dissolved oxygen level at a value of 7.9–8.1 mg/L; pH = 6.8; conductivity= 1488–1625 μS. Fishes were maintained at T = 25 °C with photoperiod 14 h light/10 h dark. Fishes were fed all the time of the experiment once a day with nauplii *Artemia salina.*

The dosing adopted for zebrafish adults was based on embryo experiments. The final exposure concentrations of the HKUST-1 suspension were 0, 0.01, 0.05, 0.1, 0.5, 1.0, 2.0, and 3.0 for zebrafish embryos, and 0, 0.5, 1.0, 2.0, 3.0, 4.0, and 5.0 mg/L for adult zebrafish. Fish mortalities were monitored at 24, 48, 72, and 96 h intervals, dead fishes were removed from the exposure tanks. At the end of the experiment, internal organs (gastrointestinal tract, liver, spleen) were removed from survived fishes to assess copper accumulation.

Statistical processing of results was carried out using the program GraphPad Prism version 5.0 (GraphPad Software, San Diego, CA, USA).

## 4. Conclusions

Nano-sized MOF, i.e., nanoHKUST-1 particles is a porous material with great potential for biomedical applications. Their environmental toxic effects are still under investigation. This study indicates that MOF’s degradation and metal escape may play a significant role in explaining the Cu-MOF toxicity.

The present work shows that differences in physiology between zebrafish forms may play the role in explaining differences in their sensitivity to Cu-containing materials, namely, nanoHKUST-1 and CuSO_4_.

## Figures and Tables

**Table 1:** Estimated and published LOEC and LC_50_ values for copper ions and HKUST-1 * for adult and embryos zebrafish.

Sample	Cu^2+^ Ions (CuSO_4_)	HKUST-1 (ὡ[Cu] = 34–35%) *, mg/L
LOEC, mg/L—embryos	0.184	0.422 mg Cu/L (1.205—HKUST-1)
LOEC, mg/L—adults	0.050–0.100 **	0.375 mg Cu/L (1.071—HKUST-1)
LC_50_, mg/L—embryos	0.396	0.746 mg Cu/L (2.132—HKUST-1)
LC_50_, mg/L—adults	0.122–0.130 **	0.525 mg Cu/L (1.500—HKUST-1)

*—Data are presented based on the copper concentration; in brackets for HKUST-1. ** literature data [31,32].
